# Up-regulation of miR-10a-5p expression inhibits the proliferation and differentiation of neural stem cells by targeting *Chl1*: Role of miR-10a-5p in NSCs

**DOI:** 10.3724/abbs.2024078

**Published:** 2024-06-05

**Authors:** Juan Zhang, Lihong Yang, Yuqing Sun, Li Zhang, Yufei Wang, Ming Liu, Xiujuan Li, Yuxiang Liang, Hong Zhao, Zhizhen Liu, Zhiyong Qiu, Ting Zhang, Jun Xie

**Affiliations:** 1 Department of Biochemistry and Molecular Biology School of Basic Medical Science Shanxi Key Laboratory of Birth Defect and Cell Regeneration MOE Key Laboratory of Coal Environmental Pathogenicity and Prevention Shanxi Medical University Taiyuan 030001 China; 2Department of Cell Biology and Genetics School of Basic Medical Science Shanxi Medical University Taiyuan 030001 China; 3 Beijing Municipal Key Laboratory of Child Development and Nutriomics Capital Institute of Pediatrics Beijing 100020 China

**Keywords:** neural tube defects, retinoic acid, miR-10a-5p, cell adhesion molecule L1-like (Chl1), ERK1/2 MAPK signaling pathway

## Abstract

Neural tube defects (NTDs) are characterized by the failure of neural tube closure during embryogenesis and are considered the most common and severe central nervous system anomalies during early development. Recent microRNA (miRNA) expression profiling studies have revealed that the dysregulation of several miRNAs plays an important role in retinoic acid (RA)-induced NTDs. However, the molecular functions of these miRNAs in NTDs remain largely unidentified. Here, we show that miR-10a-5p is significantly upregulated in RA-induced NTDs and results in reduced cell growth due to cell cycle arrest and dysregulation of cell differentiation. Moreover, the cell adhesion molecule L1-like ( *Chl1)* is identified as a direct target of miR-10a-5p in neural stem cells (NSCs) *in vitro*, and its expression is reduced in RA-induced NTDs. siRNA-mediated knockdown of intracellular *Chl1* affects cell proliferation and differentiation similar to those of miR-10a-5p overexpression, which further leads to the inhibition of the expressions of downstream ERK1/2 MAPK signaling pathway proteins. These cellular responses are abrogated by either increased expression of the direct target of miR-10a-5p ( *Chl1*) or an ERK agonist such as honokiol. Overall, our study demonstrates that miR-10a-5p plays a major role in the process of NSC growth and differentiation by directly targeting *Chl1*, which in turn induces the downregulation of the ERK1/2 cascade, suggesting that miR-10a-5p and Chl1 are critical for NTD formation in the development of embryos.

## Introduction

Neural tube defects (NTDs), including spina bifida, anencephaly, myelomeningocele and encephalocele, are severe congenital anomalies caused by incomplete or defective neural tube closure during embryonic development [ 1– 3]. NTDs are considered the second most common category of birth defects [4], affecting approximately one in every 1000 established pregnancies with a prevalence rate of 0.2–10 per 1000 in different areas [5]. The causes of NTDs appear to be complex and involve multiple genetic and environmental factors [ 6– 8]. To date, few genes have been identified as the major contributors to NTD susceptibility, along with several known environmental risk factors, such as maternal diabetes, valproic acid, folic acid and retinoic acid (RA) [9].

RA is a vitamin A-derived metabolite that is involved in embryonic development, especially in the development of the central nervous system [10], including the migration of nerve fold cells, the closure of neural tubes, and the formation of the anterior, middle and posterior brain [11]. Excessive RA level in pregnant mammals can lead to abnormalities in brain development, including NTDs [12]. At present, excessive RA-induced animal models have been widely used to study the pathogenesis of NTDs [ 13, 14].

MiRNAs are small noncoding RNAs that are involved in the posttranscriptional regulation of gene expression by binding to the 3′-untranslated regions (3′-UTRs) of target genes, causing mRNA degradation or translational inhibition. The nervous system is enriched in miRNAs, which play important roles in neural development [ 15– 18]. The possible mechanism underlying the effectiveness of environmental risk factors in NTDs was recently explored via miRNA expression using low- or high-density microarrays [ 19– 23], including reports of the abnormal expression of miRNAs in NTDs. In particular, several studies have reported that miRNA expression dysregulation contributes to the mechanism of RA-induced NTDs [ 24, 25]. The miR-10 family consists of miR-10a and miR-10b, which are highly conserved gene families whose genome is located in the vicinity of *Hox* gene developmental regulators [26]. As miR-10 has also been found to target Hox transcripts in several species, it is likely that miR-10 family members may play important roles during development [27]. It has been reported that the expression of miR-10a is significantly increased in ethanol-induced birth defects in mice, and folic acid supplementation could inhibit the occurrence of ethanol-induced defects, which is partly due to the downregulation of miR-10a expression [28]. However, it is unclear how miR-10a plays a role in neurulation in the developing neuroepithelium.

The development of mammalian neural tubes requires proper coordination of cell proliferation, adhesion, migration, differentiation and apoptosis. Thus, in mice, NTDs can be caused by exposure to antimitotic agents [29], excessive cell proliferation [30], and mutation of genes encoding proteins associated with cell cycle progression or prevention of neuronal differentiation [ 31– 33]. Li *et al*. [34] reported that miR-10a-5p inhibits chicken granulosa cell proliferation by targeting MAPRE1. Wang *et al*. [35] reported that miR-10a-5p restrains adipogenic differentiation by targeting Fasn. These studies indicated that miR-10-5p may affect the proliferation and differentiation of cells, thus affecting normal cell function. However, the effect of miR-10a-5p on NTDs remains largely unknown. Therefore, we speculated that miR-10a-5p may cause NTDs by affecting cell proliferation and differentiation.

In the present study, we used an RA-induced mouse NTD model to correlate the spatial and temporal expression patterns of miRNAs with those of NTDs. Surprisingly, we identified miR-10a-5p as a unique miRNA that is significantly overexpressed in RA-induced NTDs (approximately 50 times) by small RNA deep sequencing (sRNA-seq). We then investigated the role of miR-10a-5p in the proliferation and differentiation of neural stem cells (NSCs). To explore the underlying mechanism of miR-10a-5p in NTDs, we explored a promising target in NSCs. We identified potential target genes of miR-10a-5p using bioinformatics algorithms combined with sRNA and mRNA sequencing data integration analysis. These findings led to the prediction that *Chl1* is an important target gene of miR-10a-5p, as well as a nerve cell adhesion factor gene [36]. We identified *Chl1* as a target gene of miR-10a-5p and demonstrated that it can partially mediate the function of miR-10a-5p in NSCs. Furthermore, we preliminarily confirmed that high expression of miR-10a-5p inhibits the ERK/MAPK signaling pathway. Taken together, our results demonstrated a link between miR-10a-5p expression and NTDs, which enhanced our understanding of abnormal epigenetic modifications in NTDs.

## Materials and Methods

### Animals

C57BL/6 mice (9‒10 weeks, 18‒23 g) were obtained from Shanxi Medical University Laboratory Animal Center (Taiyuan, China) and housed under standard pathogen-free conditions at 22±2°C with 40%‒70% humidity and a 12/12-h light/dark cycle during the experiment. A standard pelleted rodent diet with tap water was provided to the mice *ad libitum*. The mating behavior of mature female mice was monitored. The effectiveness of mating was confirmed by the presence of a vaginal plug and was defined as embryonic day 0.5 (E0.5). The pregnant mice were randomly divided into control and NTD model groups. All animals were handled in strict compliance with the Guide for the Care and Use of Laboratory Animals and the Principles for the Utilization and Care of Vertebrate Animals. The Ethics Board of Shanxi Medical University approved the study protocol (animal ethics approval number: 2016LL066). All animal experiments were conducted in compliance with the guidelines of the Laboratory Animal Center of Shanxi Medical University.

**Figure 1:**
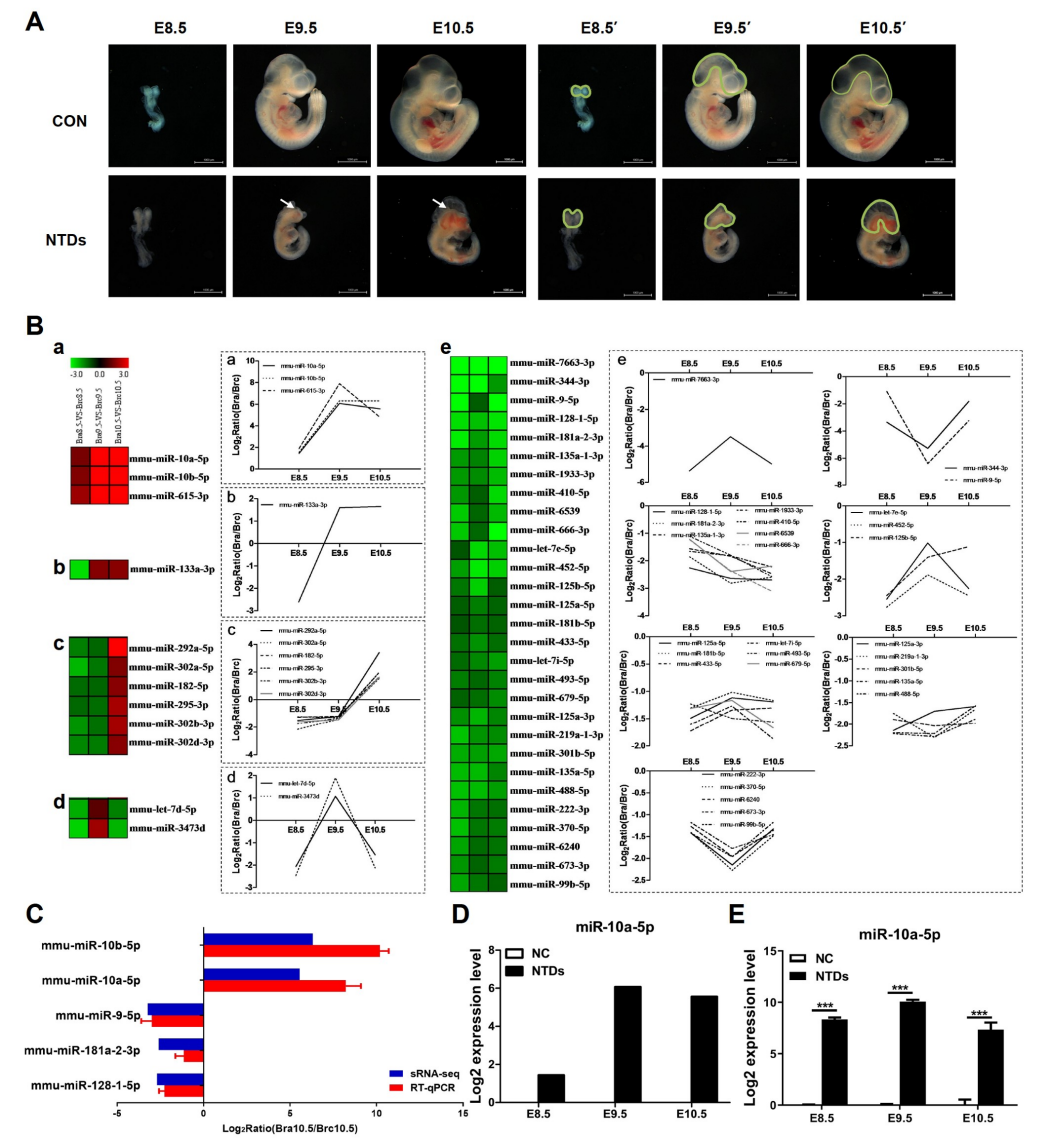
Figure 1 miR-10a-5p is significantly upregulated in RA-induced NTD mouse embryos (A) Morphology of normal and NTD embryos at E8.5, E9.5 and E10.5. Arrows indicate abnormal sections. The neural tubes demarcated by the green lines at E8.5’, E9.5’ and E10.5’ were excised from E8.5, E9.5 and E10.5 embryos, respectively. (B) Hierarchical clustering plot showing representative expression patterns of 41 DEmiRNAs during mouse neural tube development. (a‒e) These miRNAs were mainly classified into five categories. (C) RT-qPCR validation of 5 DEmiRNAs identified by sRNA-seq. (D) miR-10a-5p expression analysis of sRNA-seq results at E8.5, E9.5 and E10.5. (E) Relative expression of miR-10a-5p was measured by RT-qPCR at E8.5, E9.5 and E10.5. ***P<0.001.

At E7.5, mice in the NTD model group and control group were gavaged with 28 mg/kg (body weight) RA (Sigma, St Louis, USA) and sesame oil, respectively. On E8.5, E9.5 and E10.5, the pregnant mice were euthanized by cervical dislocation. Based on the number of somites, 10‒12, 22‒27 and 35‒39 embryos were selected on E8.5, E9.5 and E10.5, respectively [37]. Embryonic neural tubes from the most rostral aspect of the forebrain to the caudal aspect of the hindbrain were excised according to a previous study [38] and further trimmed to eliminate any nonneural tissues as precisely as possible; the precise scope is shown in Figure 1A.

### Total RNA extraction, quality control, library preparation and sRNA-seq

Nine mouse embryos were collected from three independent dams as pooled samples for each stage of the sRNA-seq experiment. Total RNA was extracted from mouse embryo NT tissue samples using Trizol reagent (Invitrogen, Carlsbad, USA) following the manufacturer’s instructions. The quality and quantity of the RNA samples were assessed on a Bioanalyzer 2100 system using an RNA 6000 Nano Reagents Port 1 kit (Agilent Technologies, Santa Clara, USA). The optical density (OD) values at 260/280 nm were confirmed to be above 1.8, and the RIN values were ≥9.6 for all RNA samples. Small RNA libraries were generated from the purified RNA using a TruSeq Small RNA Sample Preparation kit (Illumina, San Diego, USA) according to the manufacturer’s guidelines. Briefly, small RNAs ranging from 18 to 30 nt in length were gel-purified and ligated with proprietary adapters to the 5′ and 3′ termini. Reverse transcription followed by PCR was performed to obtain sufficient products for small RNA library construction and high-throughput sequencing. The cDNA libraries were then subject to single-end sequencing on an Illumina HiSeq ^TM^ 2000 platform. Base calling was performed with Illumina built-in software, and the sequences obtained from the control group were named Brc8.5, Brc9.5 and Brc10.5, while those from the NTD group were named Bra8.5, Bra9.5 and Bra10.5.

### Cell culture and transfection

Primary NSCs were isolated from mouse embryos at E12.5. Briefly, the brain vesicles of mouse embryos were collected under sterile conditions and suspended as single cells by mechanical blowing. After filtration and centrifugation, NSCs were resuspended, and 1×10 ^6^ cells/mL were added to 5 mL of DMEM/F12 medium (Gibco, Carlsbad, USA) supplemented with 2% B27 (Gibco), 20 ng/mL epidermal growth factor (EGF; PeproTech, Cranbury, USA), 20 ng/mL basic fibroblast growth factor (bFGF; PeproTech) and 1% penicillin/streptomycin solution (Pen/Strep). After incubation at 37°C under a 5% CO _2_ atmosphere for 5 days, NSCs formed large spherical colonies (passage 0 neurospheres). The cells were passaged every 4 days, and the cells were passaged at least twice before they were used for subsequent experiments. To investigate NSC differentiation, passage 2 neurospheres were collected, dissociated into single cells, and stimulated with differentiation medium (DMEM/F12) supplemented with 5% fetal bovine serum (Gibco) without growth factors. The medium was changed every 3 days, and the cells were cultured for 7 days at 37°C. The human embryonic kidney cell line 293T (HEK293T; ATCC, Manassas, USA) was cultured in Dulbecco’s modified Eagle’s medium (DMEM; Sigma) supplemented with 10% fetal bovine serum and 1% Pen/Strep.

mmu-miR-10a-5p and mouse *Chl1* were cloned and inserted into the LVX-hEF1a-EGFP-gene-PGK-PURO and pAD-mCMV-GFP-MCS-3FLAG vectors, respectively. The constructs were sent to Sunbio Medical Biotechnology (Shanghai, China) for lentivirus and adenovirus packaging and purification. NSCs were injected with either 40 moi LV-miR-10a or LV-NC and AV-Chl1 or control viruses (AV-NC). siRNA against Chl1 (siR-Chl1) (siRNA-1: 5′-CCACATCTCTCACTTTCAA-3′; siRNA-2: 5′-GCTACAAAGCTCAGAGTTT-3′; siRNA-3: 5′-GGTCCAAGCCATCAATCAA-3′) and siR-NC (Cat No. siN0000001-1-5; https://www.ribobio.com/product/siN0000001-1-5/) were synthesized by RiboBio Co., Ltd. (Guangzhou, China). After NSCs were dissociated into single cells, these oligonucleotides were transfected into NSCs at a final concentration of 100 nM using HiPerFect Transfection Reagent (Qiagen, Hilden, Germany) according to the manufacturer’s protocol. After 48 h of incubation, RNA and total cellular protein were extracted from the cells and subjected to RT-qPCR and western blot analysis, respectively.

In addition, 24 h after LV-miR-10a infection, the NSCs were treated by honokiol (MedChemExpress, Shanghai, China) to activate p-ERK1/2. After 48 h of incubation, the total cellular protein was extracted from the cells and subjected to western blot analysis. BrdU assay was performed after 24 h incubation, and immunocytochemistry was performed after 7 days incubation.

### Quantitative real-time PCR of miRNA (TaqMan) and mRNA

Total RNA, including the small RNA fraction, was extracted using a mirVana miRNA Isolation kit (Ambion, Austin, USA) according to the manufacturer’s protocol. Reverse transcription of 5 μL of eluate in a 15 μL reaction mixture was carried out using a TaqMan ^®^ MicroRNA Reverse Transcription kit (Applied Biosystems, Foster City, USA) according to the manufacturer’s instructions. The standard MicroRNA TaqMan ^®^ tests (Applied Biosystems) used for miRNA quantification were as follows: mmu-miR-10a-5p (TM: 000387), mmu-miR-128-1-5p (TM: 464067), mmu-miR-181a-2-3p (TM: 002687), mmu-miR-9-5p (TM: 000583), mmu-miR-10b-5p (TM: 002218) and U6 snRNA (TM: 001973) as a control. A 20-μL reaction mixture was prepared for quantitative PCR containing 1.33 μL of the product from the RT reaction, 10 μL of TaqMan Universal PCR Master Mix, 1 μL of TaqMan miRNA assay (20×) and nuclease-free water. The reaction was carried out with a 10-min incubation at 95°C followed by 40 cycles of 95°C for 15 s and 60°C for 1 min.

For mRNA, total RNA from cells was extracted using Trizol and reverse-transcribed into cDNA with a RevertAid First Strand cDNA Synthesis kit (Thermo Fisher Scientific, Waltham, USA) according to the manufacturer’s instructions. qPCR was performed on a Step-One Real-Time PCR System (Applied Biosystems) using SYBR Green qPCR Master Mix (TaKaRa, Dalian, China). All mRNA primer sequences were provides in Supplementary Table S1.

All reactions were run in triplicate, and the expression levels of miRNA and mRNA were calculated using the ΔCT method. The *U6* snRNA level was used as an internal normalization control for miRNAs and the *Actb* mRNA level was used as an internal normalization control for mRNAs.

### BrdU assay

Bromodeoxyuridine (BrdU) was added to the culture medium at a final concentration of 10 μM. After 24 h of culture, the number of BrdU-positive cells was measured by immunofluorescence microscopy. Briefly, cells were cultured in 24-well plates treated with polylysine. After fixation with paraformaldehyde, the cells were pretreated with 2 M HCl to denature the DNA and then incubated with a mouse anti-BrdU antibody (1:1000; Abcam, Cambridge, UK) at 4°C overnight. The plate was placed under a fluorescence microscope, and BrdU-positive cells were counted in at least 20 microscopic fields. The total number of cells in each microscopic field was determined by DAPI (Solarbio, Shanghai, China) staining.

### Cell cycle analysis

After two days of transfection, the cells were collected, fixed with 70% cold ethanol at 4°C for 24 h and stained with 50 μg/mL propidium iodide (PI; Keygen, Shanghai, China) for 30 min. The cell cycle distribution was analyzed on a Coulter Epics XL flow cytometer (BD Biosciences, Franklin Lakes, USA), and the number of cells in distinct cell cycle phases was determined using ModFit LT3.2 (Verity Software House, Augusta, USA).

### Immunocytochemistry

NSCs were identified using standard immunocytochemical staining for Nestin, while differentiated NSCs were identified by staining for GFAP, GALC, NF-H and MAP2, which are markers of astrocytes, oligodendrocytes and neurons, respectively. Cells were fixed with paraformaldehyde (4%, w/v) in PBS and then permeabilized with 0.1% Triton X-100 (Sigma) at room temperature for 10 min. After being blocked with 5% BSA for 1 h, the cells were washed and incubated with primary rabbit anti-Nestin polyclonal antibody (1:500 dilution; Bioss, Beijing, China), primary rabbit anti-GFAP polyclonal antibody (1:200 dilution; Bioss), primary rabbit anti-GALC polyclonal antibody (1:200 dilution; Absin, Shanghai, China), primary rabbit anti-NF-H polyclonal antibody (1:200 dilution; Bioss), and rabbit anti-MAP2 monoclonal antibody (1:500 dilution; Abcam) at 4°C overnight. After three washes, the cells were incubated with a goat anti-rabbit IgG-Cy3 secondary antibody (1:200 dilution; Absin) at room temperature for 2 h. The nuclei were counterstained with DAPI at room temperature for 10 min. The samples were visualized with a fluorescence phase contrast inverted microscope (Nikon, Tokyo, Japan).

### Luciferase reporter assay

The *Chl1* 3′-UTR-WT and 3′-UTR-MUT gene fragments were inserted into the pmirGlo ^®^ Dual Luciferase miRNA target expression vector by GenePharma Co., Ltd. (Shanghai, China). For luciferase assays, HEK293T cells were co-transfected in 96-well plates with 200 ng of pmirGlo ^®^ Dual Luciferase miRNA target expression vector and miR-10a-5p mimics or non-targeting siRNA control (NC) at a final concentration of 50 nM using Lipofectamine ^®^ 2000 (Thermo Fisher Scientific) according to the manufacturer’s instructions. Luciferase activities were measured with a Dual-Glo ^®^ Luciferase Assay System (Promega, Madison, USA) after 24 and 48 h, and *Renilla luciferase* activity was normalized to Fluc activity.

### Western blot analysis

To extract proteins from tissue, we used embryonic brain neural tube tissue from twenty pregnant mice. Total cellular/tissue proteins were extracted using radioimmunoprecipitation assay (RIPA) buffer (Solarbio) supplemented with 1% PMSF and 1% phosphatase inhibitor. The protein concentration was quantified using BCA (Minibio, Shanghai, China) according to the manufacturer’s instructions. Equal amounts of protein were separated by 10% SDS-PAGE and transferred to a PVDF membrane (Millipore, Billerica, USA). The membrane was blocked in 5% milk TBS supplemented with 0.1% Tween-20 and then incubated with antibodies against Chl1 (1:800 dilution; R&D, Minneapolis, USA), phospho-SRC (1:1000 dilution; CST, Beverly, USA), total SRC (1:1000 dilution; CST), phospho-PI3K (1:1000 dilution; CST), total PI3K (1:1000 dilution; CST), phospho-ERK1/2 (1:1000 dilution; CST), total ERK1/2 (1:1000 dilution; CST) and GAPDH (1:1000 dilution; Santa Cruz, Santa Cruz, USA) overnight. The membranes were washed and incubated with HRP-conjugated AffiniPure goat anti-rabbit IgG or HRP-conjugated AffiniPure rabbit anti-goat IgG (1:5000 dilution; Boster, Wuhan, China). Immunoreactive bands were visualized by chemiluminescence using enhanced chemiluminescence (ECL) western blotting substrate (Solarbio) in a ChemiDoc high-sensitivity chemiluminescence imaging system (Bio-Rad, Hercules, USA). The relative intensities of the protein bands were analyzed using ImageJ software (NIH, Bethesda, USA).

### Immunohistochemical staining and immunofluorescence assay

Immunohistochemical analyses were performed on six paraffin-embedded mouse embryo brain tissues collected from six independent dams at E10.5 to detect the localization of Chl1-positive cells. Briefly, sections were treated with 3% hydrogen peroxide for 20 min to block endogenous peroxidase activity. For antigen retrieval, the slides were placed in racks containing EDTA buffer (pH 9.0) and heated in a microwave oven at maximum power for 10 min. After that, the sections were incubated with primary antibodies against Chl1 (1:100 dilution; R&D) at 4°C overnight. Then, the slides were incubated with an Alexa Fluor 647-conjugated secondary antibody (1:500 dilution; Invitrogen) at room temperature for 1 h. The sections were counterstained with DAPI to identify the nuclei and then mounted with Permount. Photomicrograph images were captured using a fluorescence phase contrast inverted microscope (Nikon) with Picture Frame software.

### Statistical analysis

All the statistical analyses were performed with GraphPad Prism 8.0 software (GraphPad Software, La Jolla, USA). Data are expressed as the mean±SEM. Two-tailed Student’s *t* tests were performed to analyze the differences between two groups. Comparisons of parameters among 3 or more groups were performed using one-way ANOVA with a post hoc test for multiple testing corrections. All procedures were performed in triplicate. *P*<0.05 were considered to indicate statistical significance.

## Results

### RA-induced mouse NTD embryo model

RA-induced NTDs have been widely adopted as reliable and convenient NTD models [ 39– 41]. In this study, we used a modified rapid RA-induced NTD mouse model via excess RA gavage (28 mg/kg) at E7.5, as described in our previous studies [ 24, 42]. The results of the RA-induced NTD mouse model are presented in Supplementary Table S2. Treatment with RA was overwhelmingly teratogenic, resulting in 78% of the mouse embryos showing NTDs. Almost all the embryos with NTD phenotypes were developmentally delayed, which was consistent with the findings of a previous study [12].

As shown in Figure 1A, at E8.5, the embryos in the control group exhibited a ″U″ shape, with the body axis not yet turned. The head nerve folds were extended toward the rostral side to form a shallow ″V″-shaped nerve groove. However, the closure of nerve folds in the NTD group was delayed. On E9.5, the embryos in the control group formed a ″C″ shape, and their brains developed normally. The nerve folds fused in the dorsal midline to form the neural tube. Malformed embryos in the NTD group were significantly developmentally delayed, and the neural tube was not closed. On E10.5, the embryonic development of mice in the control group was normal, with a smooth, rounded and plump brain and a completely closed neural tube. In contrast, malformed embryos in the NTD group showed delayed development and failed closure of the neural tube, possibly accompanied by enlarged heart and ventricular chambers, short tails, and unfinished turning of the neural axis [42].

### MiR-10a-5p is highly expressed in the brains of NTD embryos

To define alterations in the miRNA expression profile accompanying RA-induced morphological changes, sRNA-Seq was performed on mouse neural tube tissue samples from normal and RA-treated E8.5, E9.5 and E10.5 embryos. Nine mouse embryos were collected from three independent dams as pooled samples for each stage of the sRNA-seq experiment. After RNA extraction, cDNA library construction and Illumina sequencing, three control miRNA libraries (Brc8.5, Brc9.5 and Brc10.5) and three NTD miRNA libraries (Bra8.5, Bra9.5 and Bra10.5) were obtained. In the Bra8.5-vs-Brc8.5, Bra9.5-vs-Brc9.5, and Bra10.5-vs-Brc10.5 library comparisons, 241, 271 and 213 differentially expressed miRNAs (DEmiRNAs) were identified, respectively [43]. Then, intersection analysis among Bra8.5-vs-Brc8.5, Bra9.5-vs-Brc9.5 and Bra10.5-vs-Brc10.5 was performed, and 41 coexpressed DEmiRNAs were obtained ( Supplementary Table S3). Furthermore, we conducted hierarchical clustering analysis of 41 DEmiRNAs across different time points. As shown in Figure 1B, the subsets of miRNAs were clustered into five categories as follows. (1) Group a: miRNAs in the NTD group were upregulated at all three time points compared with those in the control group. There are three miRNAs belonging to this category, namely, miR-10a-5p, miR-10b-5p and miR-615-3p ( Figure 1B, a). (2) Group b: miRNAs, such as miR-133a-3p, in the NTD group were downregulated at E8.5 but upregulated at E9.5 and 10.5 compared with those in the control group ( Figure 1B, b). (3) Group c: miRNAs in the NTD group, including miR-292a-5p, miR-302a-5p, miR-182-5p, miR-295-3p, miR-302b-3p and miR-302d-3p, were downregulated at E8.5 and E9.5 but upregulated at E10.5 compared with those in the control group ( Figure 1B, c). (4) Group d: miRNAs in the NTD group, such as let-7d-5p and miR-3473d, were downregulated at E8.5 and E10.5 but upregulated at E9.5 compared with those in the control group ( Figure 1B, d). (5) Group e: the downregulated miRNA clusters in which the expression levels of miRNAs, including miR-7663-3p, miR-344-3p, miR-9-5p, miR-128-1-5p and miR-181a-2-3p, were consistently decreased in the NTD group ( Figure 1B, e).

To confirm these results obtained from sRNA-seq, RT-qPCR was performed. As shown in Figure 1C, the expressions of miR-10b-5p and miR-10a-5p were upregulated, while the expressions of miR-9-5p, miR-181a-2-3p and miR-128-1-5p were downregulated in mice with RA-induced NTDs. The expression patterns of these five miRNAs determined by RT-qPCR were similar to those determined by deep sequencing, indicating the reliability of the deep sequencing data. However, the fold change in expression obtained by RT-qPCR was slightly different from the results of deep sequencing analysis, which may be caused by differences in the sensitivity, specificity, and algorithm between these two techniques.

Compared with the normal control group, miR-10a-5p was identified as one of the most significantly upregulated miRNAs in NTD embryos by sRNA-seq ( Figure 1D). We further examined miR-10a-5p expression levels in normal and NTD embryo tissues at E8.5, E9.5 and E10.5 by RT-qPCR. Similarly, the expression of miR-10a-5p was found to be greater in NTD tissues than in normal mouse embryos ( Figure 1E). These results suggested that miR-10a-5p may play an important role in RA-induced brain abnormalities.

### Overexpression of miR-10a-5p inhibits NSC cell growth and differentiation

The primary cultured NSCs grew into pellets, and immunocytochemistry results showed that the neurospheres expressed Nestin ( Supplementary Figure S1). The results of differentiation identification showed that NSCs could differentiate into neurons, astrocytes, and oligodendrocytes ( Supplementary Figure S2). These results indicated that the method used for the isolation and culture of NSCs was appropriate and that the neurospheres were composed mainly of NSCs with strong cell stemness, laying the foundation for cell culture for experimental research.

**Figure 2:**
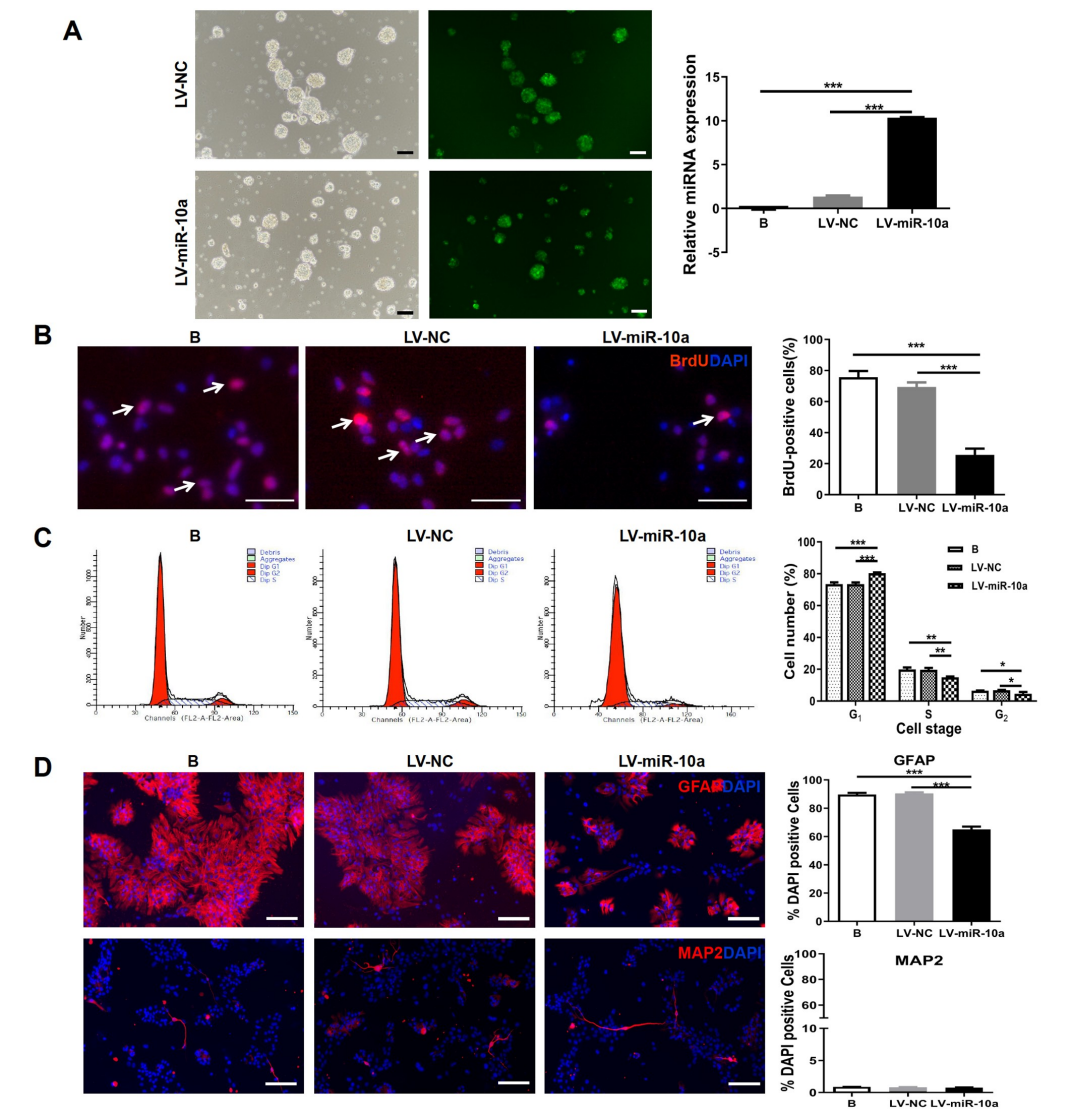
Figure 2 Effects of miR-10a-5p overexpression on cell proliferation and differentiation (A) Representative micrographs of GFP expression in NSCs after transfection with LV-NC or LV-miR-10a for 72 h and RT-qPCR results showing the relative expression of miR-10a-5p. (B) Representative micrographs of immunofluorescence staining for BrdU (red) with nuclei stained blue with DAPI. Arrows indicate BrdU+ cells. The percentage of BrdU+ cells was significantly lower in the LV-miR-10a group than in the blank or LV-NC groups. (C) FCM results of the cell cycle analysis are shown. The percentage of cells in the G1 phase was significantly greater and the percentage of cells in the S and G2 phases was significantly lower in the LV-miR-10a group than in the blank or LV-NC groups. (D) Representative micrographs of immunofluorescence staining for GFAP or MAP2 (red) with nuclei stained blue with DAPI. The percentage of GFAP+ cells was significantly lower in the LV-miR-10a group than in the blank or LV-NC groups, and no significant difference was found in the percentage of MAP2+ cells. Scale bar: 50 μm. *P<0.05; **P<0.01; ***P<0.001.

To further explore the possible role of miR-10a-5p in neural development, we constructed a highly specific miR-10a-expressing lentivirus overexpressing miR-10a-5p (LV-miR-10a) and, in parallel, a nontargeting control lentivirus (LV-NC). As shown in Figure 2A (left panel), NSCs transduced with the above-mentioned recombinant lentiviruses expressed a significant amount of green fluorescent protein (GFP) after 72 h of lentivirus transfection. RT-qPCR results indicated that compared with those in the blank group and the LV-NC group, the miR-10a-5p expression level in the group transduced with LV-miR-10a in NSCs was substantially elevated ( Figure 2A, right panel). Our results showed that the recombinant miR-10a-5p overexpression lentivirus could be transduced and expressed well in NSCs and could be used for subsequent experiments.

To study miR-10a-5p function in NTDs, cell proliferation of the above-mentioned miR-10a-5p-overexpressing NSCs was measured by a BrdU assay. Interestingly, fewer BrdU-positive (BrdU ^+^) cells were detected in the LV-miR-10a group (25.67%±2.33%) than in the blank control group (75.67%±2.33%; *P*<0.001) and the LV-NC group (69.33%±1.76%; *P*<0.001), while no significant difference was detected between the blank and negative controls ( Figure 2B). Furthermore, the cell cycle distribution of NSCs treated with LV-miR-10a was analyzed by flow cytometry (FCM). Clearly, miR-10a-5p overexpression induced cell cycle arrest at the G _1_ phase ( Figure 2C), indicating that miR-10a-5p may control the sustained growth of cells by regulating the cell cycle machinery.

Furthermore, the effects of miR-10a-5p on the differentiation of NSCs were explored using immunocytochemical staining for GFAP and MAP2. The results revealed that the relative quantity of GFAP-positive (GFAP ^+^) cells in the LV-miR-10a group (65.00%±2.08%) decreased compared with that in the blank control group (89.67%±1.20%; *P*<0.001) and LV-NC group (90.33%±0.88%; *P*<0.001), and no significant difference was observed between the blank group and LV-NC group ( *P*>0.01). Surprisingly, the proportion of neurons (MAP2 ^+^ cells/total cells) was not significantly different among the three groups ( Figure 2D). These results collectively suggested that abnormal NSCs in the neural tube of NTD patients might be due to impaired proliferation and differentiation of NSCs after increased miR-10a-5p expression.

### MiR-10a-5p directly targets *Chl1*

To explore the underlying mechanisms by which miR-10a-5p suppresses cell growth and differentiation, we screened the predicted target genes of miR-10a-5p in NTDs via the following steps. First, a combined analysis of sRNA-seq and mRNA-seq data was performed by MAGIA ^2^, which was used to analyze the target predictions and miRNA and gene expression data [ 44, 45], and 67 predicted target genes were obtained ( Supplementary Table S4). Second, based on the mRNA-seq results, gene expression in neural tubes from normal and RA-treated embryos at E8.5, E9.5 and E10.5 was filtered, and the fold change for each of these 67 genes was calculated. Among these 67 genes, compared with those in the normal group, the expressions of 46 genes in the RA-treated group did not significantly differ at any of the three time points, the expressions of 12 genes in the RA-treated group increased at least at one time point, and the expressions of 9 genes in the RA-treated group decreased at least at one time point. Considering that the expression of miR-10a-5p increased in the RA treatment group and that miRNAs mainly inhibit the expression of target genes, we screened 8 ( *Rgs8*, *Has3*, *Elavl3*, *Lhfpl4*, *Rorb*, *Gabrb2*, *Irs4* and *Chl1*) of the above-mentioned 9 genes whose expressions decreased in the RA-treated group at least at one time point as candidate prediction target genes ( Supplementary Table S4). Third, to further verify the mRNA-seq results, RT-qPCR was used to detect the expressions of these 8 genes in the neural tube tissues of normal and RA-treated mice at E10.5. The results showed that the expressions of all 8 genes in the RA-treated group were downregulated compared with those in the normal group ( Supplementary Figure S3). The expression levels of *Rgs8*, *Elavl3*, *Gabrb2*, *Irs4* and *Chl1* decreased significantly, which was consistent with the sequencing results ( Supplementary Table S4). Among these 5 predicted targets, we focused on *Chl1* because it encodes a cell adhesion molecule closely related to L1, and is highly expressed in the central and peripheral nervous systems and plays an important role in brain development and function [46]. In addition, miR-10a has been reported to target *Chl1* in human cervical cancer cells, promoting cell growth, migration, and invasion [47]. Therefore, we hypothesized that *Chl1* might play a role as a target gene of miR-10a-5p in the pathogenesis of NTDs.

**Figure 3:**
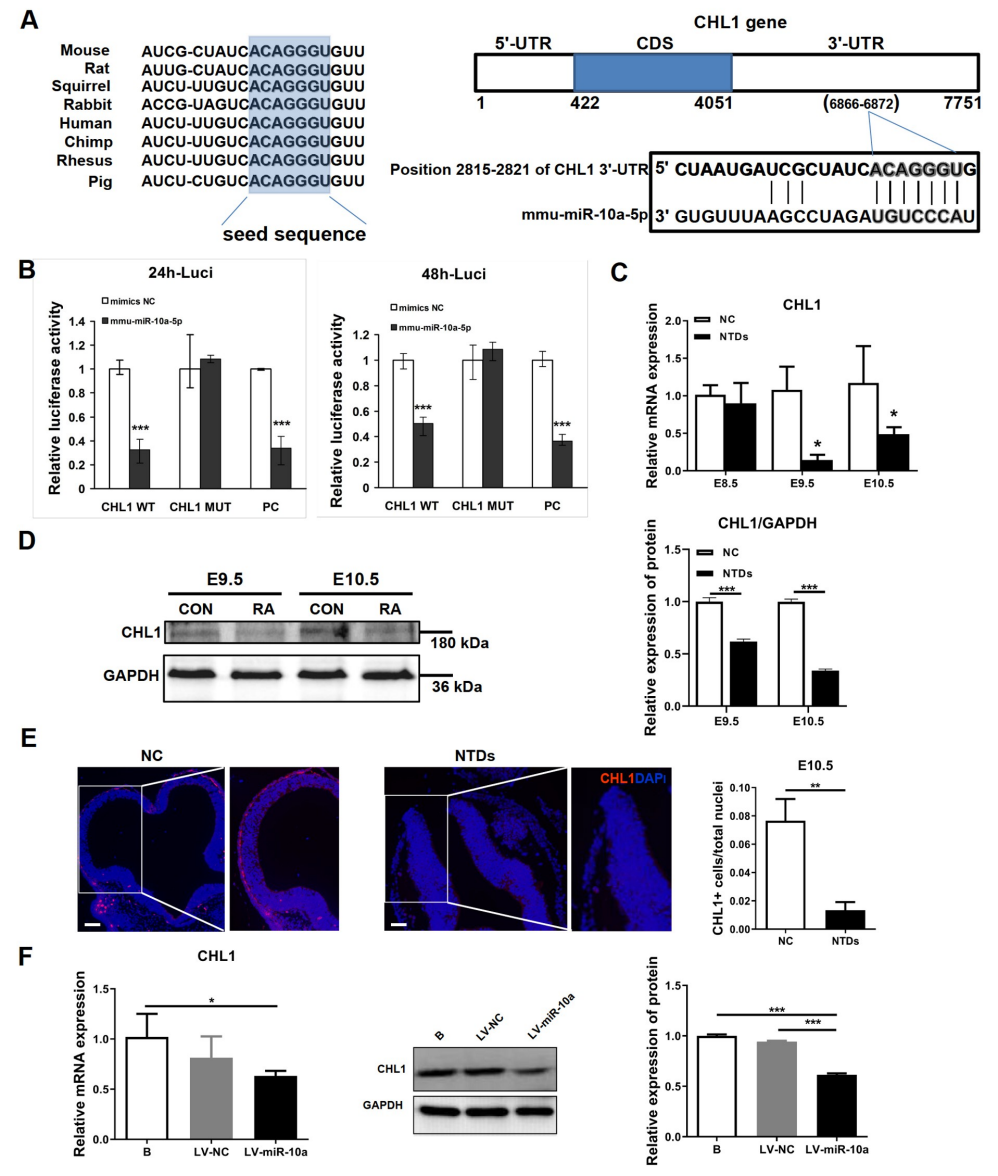
Figure 3 *Chl1* is a direct target of miR-10a-5p (A) The public miRNA databases (TargetScan) predict that Chl1 may be a target for miR-10a-5p and that the 3′-UTR of Chl1 mRNA contains a highly conserved and complementary sequence between positions 2815 and 2821 for the binding of the seed sequence of miR-10a-5p. (B) Cells were transfected with pMIR-Chl1-WT, pMIR-Chl1-MUT or miR-10a-5p positive control (PC) reporter plasmids together with miR-10a-5p mimics or negative controls. The levels of firefly and Renilla luciferase activities were assayed 24 and 48 h later. (C) Relative expression of Chl1 mRNA was measured by RT-qPCR using β-actin as an internal control in NTD tissues and normal tissues (NCs) at E8.5, E9.5 and E10.5. (D) The expression of the Chl1 protein in NTD and NC tissues at E9.5 and E10.5 was measured by western blot analysis. (E) Representative micrographs of immunofluorescence staining for Chl1 (red) with nuclei stained blue with DAPI. Areas of Chl1-positive cells were quantified by using ImageJ. (F) The expression of Chl1 mRNA was measured by RT-qPCR using β-actin as an internal control in NSCs after transfection with LV-miR-10a or LV-NC for 48 h (left). Relative Chl1 protein expression in NSCs after transfection with LV-miR-10a or LV-NC for 72 h was measured by western blot analysis using GAPDH as an internal control (right). Scale bar: 50 μm. *P<0.05; **P<0.01; ***P<0.001.

As shown in Figure 3A, there is a miR-10a-5p binding site at nt 2815-2821 of the *Chl1* 3′-UTR, which is highly conserved among different species according to comparisons of human sequences with interspecies homology. To further validate the direct binding and targeting of miR-10a-5p, we constructed *Renilla luciferase* reporters containing either the wild-type (WT) or mutated 3′-UTR of the target genes (3′-UTR-MUT). Mutations were designed within the miR-10a-5p seed-binding regions of the 3′-UTR to disrupt the predicted binding sites. Reporter activities were quantified and normalized to nontargeted firefly luciferase (Fluc) activity. *Chl1* reporters containing 3′-UTR-WT showed approximately 70% and 50% repression in response to inhibition of miR-10a-5p at 24 and 48 h, respectively ( Figure 3B), while the mutations within the miR-10a-5p binding sites of the *Chl1* 3′-UTR partially abolished the responsiveness of the corresponding reporters to miR-10a-5p, indicating that miR-10a-5p regulates the *Chl1* gene via its 3′-UTR.

Then, we further verified the expression level of Chl1 in normal and NTD embryonic tissues. RT-qPCR and western blot analysis revealed that compared with those in the control group, the mRNA and protein expression levels of Chl1 in RA-induced NTD embryonic tissues at E9.5 and E10.5 were significantly decreased ( Figure 3C,D). In addition, tissue immunofluorescence showed the expression of Chl1 in transverse sections of normal and NTD mouse embryonic hindbrain tissue at E10.5. Chl1 staining revealed red fluorescence and the nuclei were stained blue with DAPI. As shown in Figure 3E, the expression of Chl1 was greater in normally closed neural tube epithelial cells than in NTD tissues with failed neural tube closure ( *P*=0.0026).

To test whether miR-10a-5p expression affects endogenous Chl1 expression, LV-miR-10a or LV-NC was transfected into NSCs. Chl1 mRNA and protein expression levels were decreased in the LV-miR-10a group compared with those in the blank control and LV-NC groups ( Figure 3F). These results indicated that overexpression of miR-10a-5p in NSCs inhibited the expression of *Chl1*, which is a direct target of miR-10a-5p.

### *Chl1* inhibition leads to a reduction in cell growth and differentiation

**Figure 4:**
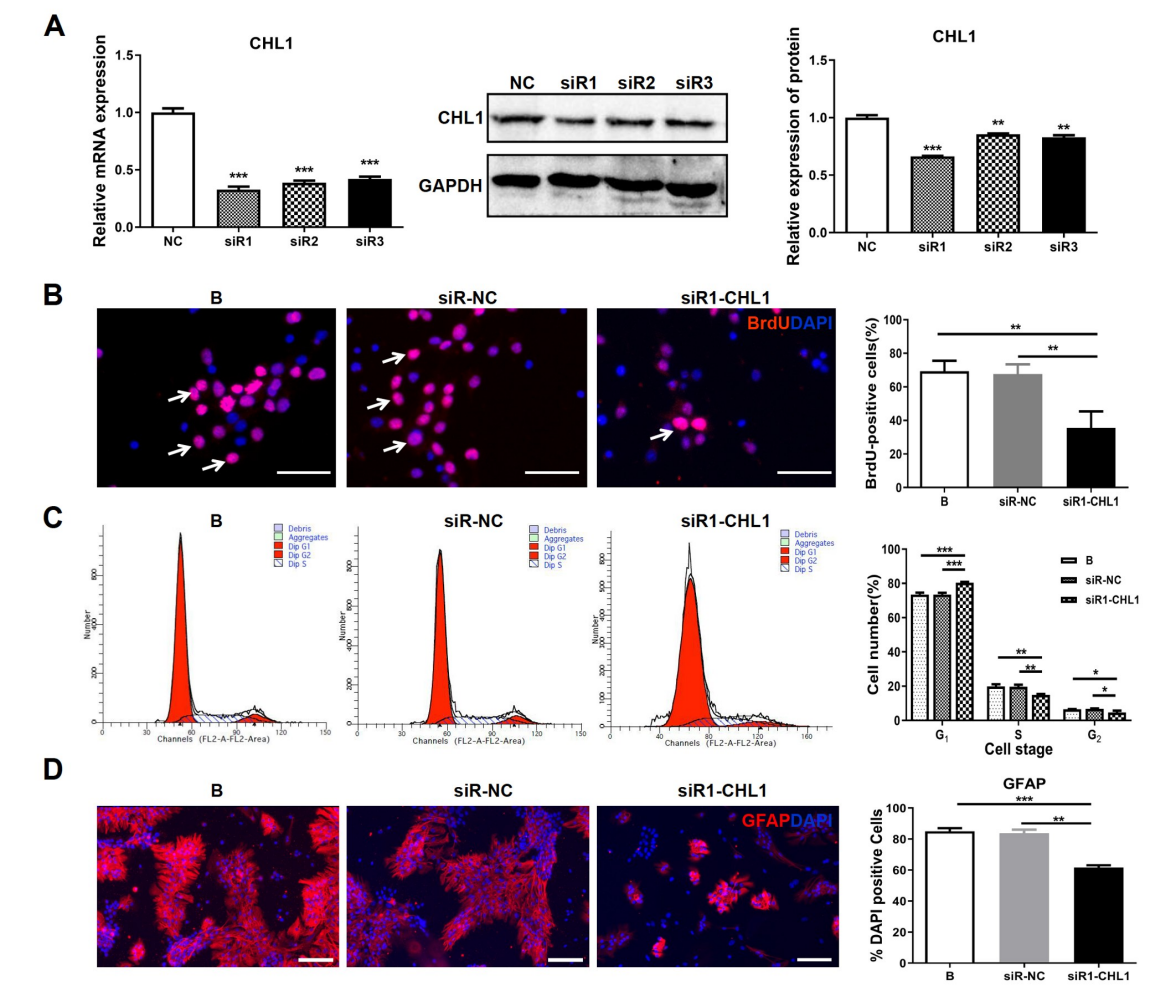
Figure 4 Effects of *Chl1* knockdown on cell proliferation and differentiation (A) The expressions of Chl1 mRNA and protein in NSCs were measured by RT-qPCR (left) and western blot (right) analysis after transfection with siR-NC, siR1-Chl1, siR2-Chl1 or siR3-Chl1 for 72 h. (B) Representative micrographs of immunofluorescence staining for BrdU (red) with nuclei stained blue with DAPI. Arrows indicate BrdU+ cells. The percentage of BrdU+ cells was significantly lower in the siR1-Chl1 group than in the blank or siR-NC groups. (C) FCM results of the cell cycle analysis are shown. The percentage of cells in the G1 phase was significantly greater and the percentage of cells in the S and G2 phases was significantly lower in the siR1-Chl1 group than in the blank or siR-NC groups. (D) Representative micrographs of immunofluorescence staining for GFAP (red) with nuclei stained blue with DAPI. The percentage of GFAP+ cells was significantly lower in the siR1-CHL1 group than in the blank or siR-NC groups. Scale bar: 50 μm. *P<0.05; **P<0.01; ***P<0.001.

To explore the function of Chl1 in NSCs, the expression of *Chl1* was downregulated by small interfering RNA (siRNA). As shown in Figure 4A, the Chl1 mRNA and protein levels were significantly lower in the siRNA-treated groups than in the siRNA control (siR-NC) group. A BrdU assay was performed to determine the impact of *Chl1* knockdown on the growth of NSCs. After transfection, the percentage of BrdU ^+^ cells in the siR1-Chl1-treated group (35.50%±4.94%) was significantly lower than that in the blank (69.25%±3.15%; *P*=0.0012) and siR-NC groups (67.50%±2.96%; *P*=0.0014) ( Figure 4B).

Cell cycle analysis by FCM was performed to determine whether *Chl1* gene silencing affects the cell cycle distribution of NSCs. Compared with those in the blank control and siR-NC groups, *Chl1* knockdown in NSCs (siR1-Chl1) caused the accumulation of cells in the G _1_ phase at 48 h post-transfection ( Figure 4C). These results suggested that knockdown of *Chl1* caused NSC cell cycle arrest in the G _1_ phase.

After 7 days of incubation, the relative quantity of GFAP ^+^ cells was measured ( Figure 4D). A significant decrease in the percentage of GFAP ^+^ cells was detected in the siR1-Chl1-treated group (61.67%±1.45%) compared with that in the blank (85.00%±2.08%; *P*<0.001) or siR-NC group (83.67%±2.40%; *P*=0.0014) ( Figure 4D). These findings suggested that Chl1 is strongly associated with NSC differentiation, which is similar to that induced by miR-10a-5p overexpression.

### Overexpression of *Chl1* reverses the effect of miR-10a-5p on the proliferation and differentiation of NSCs

**Figure 5:**
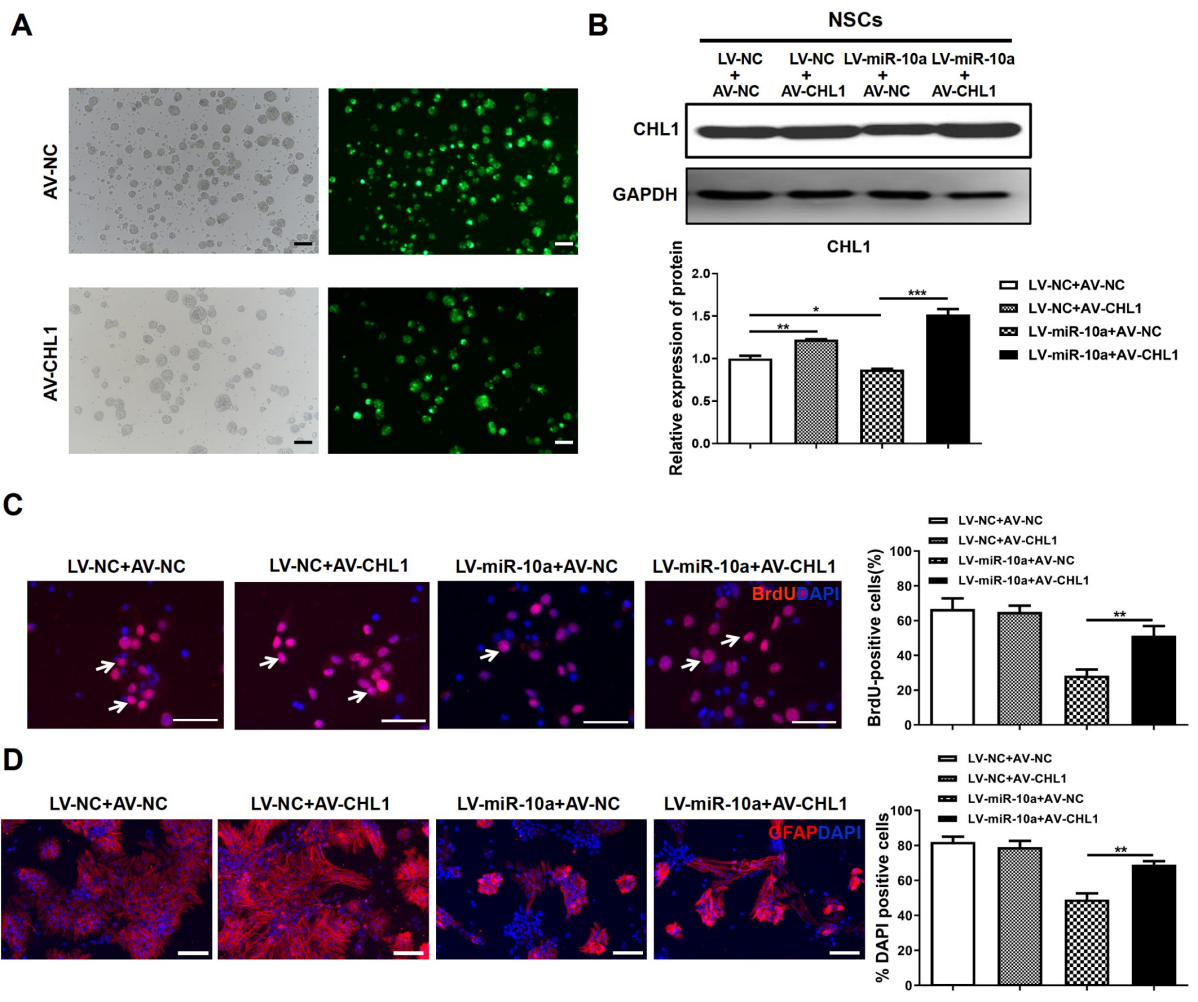
Figure 5 Re-expression of *Chl1* reverses the effect of miR-10a-5p on proliferation and differentiation of NSCs (A) Representative micrographs of GFP expression in NSCs after transfection with AV-NC or AV-Chl1 for 72 h. (B) The expression of Chl1 protein in NSCs after transfection (AV-NC or AV-Chl1) or cotransfection (LV-miR-10a or LV-NC) for 72 h was measured by western blot analysis. (C) Representative micrographs of immunofluorescence staining for BrdU (red) with nuclei stained blue with DAPI. Arrows indicate BrdU+ cells. The percentage of BrdU+ cells was significantly greater in the LV-miR-10a+AV-Chl1 group than in the LV-miR-10a+AV-NC group. The miR-10a-5p-induced proliferation of NSCs was restored via Chl1 re-expression. (D) Representative micrographs of immunofluorescence staining for GFAP (red) with nuclei stained blue with DAPI. The percentage of GFAP+ cells was significantly greater in the LV-miR-10a+AV-Chl1 group than in the LV-miR-10a+AV-NC group. The miR-10a-5p-induced differentiation of NSCs was restored by Chl1 re-expression. Scale bar: 50 μm. *P<0.05, **P<0.01; ***P<0.001.

The expression of Chl1 was restored by transfecting Chl1-expressing adenovirus (AV-Chl1) containing a *Chl1* ORF lacking the 3ʹ-UTR sequence into NSCs after 24 h of transfection with LV-miR-10a. More than 80% of NSCs expressed GFP, and the protein expression of Chl1 was upregulated significantly in AV-Chl1-infected NSCs ( Figure 5A,B). BrdU and immunofluorescence assays showed that compared with the negative control, *Chl1* overexpression had no significant effect on the proliferation or differentiation ability of NSCs. However, cotransfection of AV-Chl1 with LV-miR-10a significantly reversed LV-miR-10a-induced NSC proliferation and differentiation ( Figure 5C,D). These results suggested that miR-10a-5p acts via suppression of *Chl1* expression.

### MiR-10a-5p inhibits cell growth and differentiation through the ERK1/2 MAPK signaling pathway

**Figure 6:**
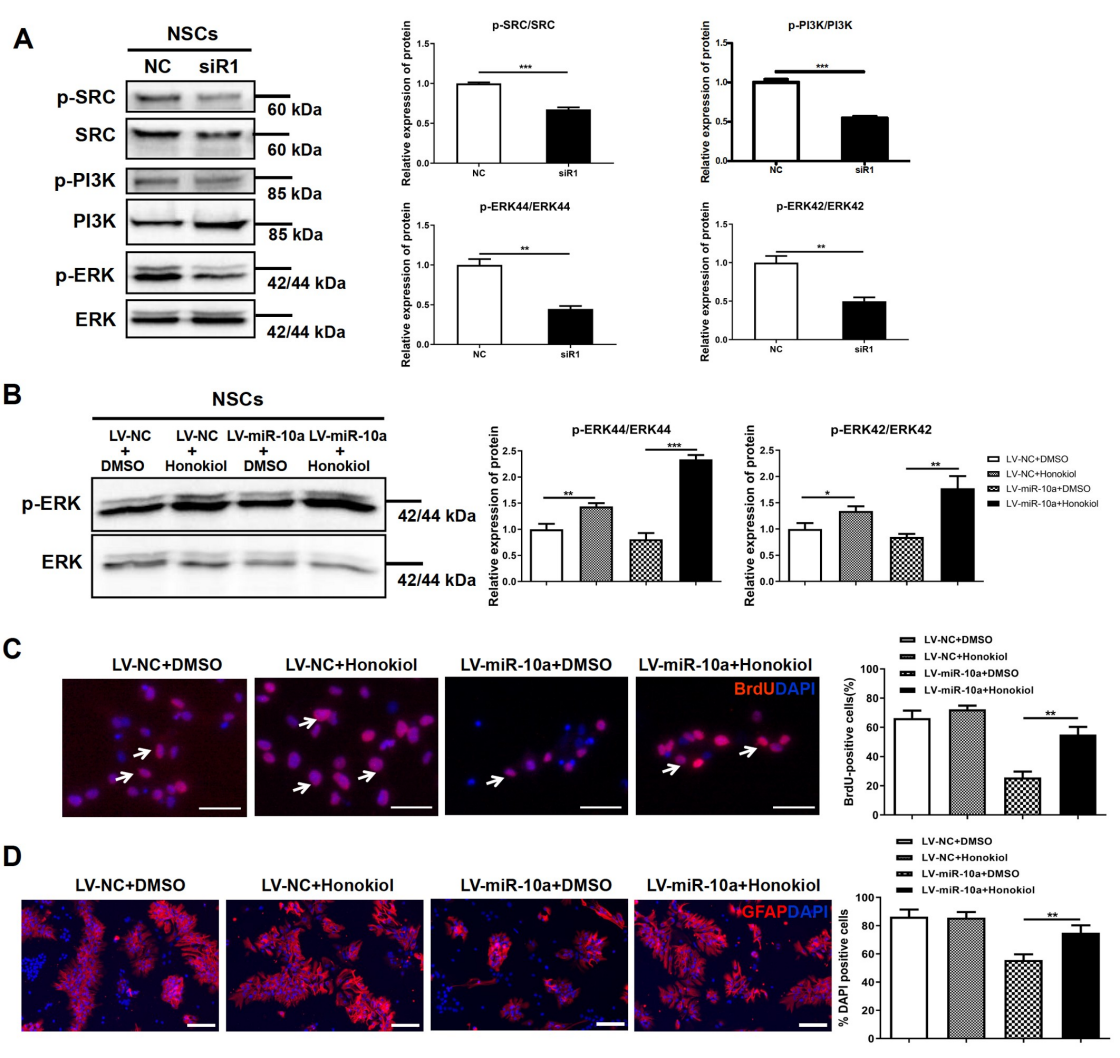
Figure 6 The ERK agonist Honokiol reverses the effects of miR-10a-5p on proliferation and differentiation of NSCs (A) The protein expression levels of p-SRC, SRC, p-PI3K, PI3K, p-ERK and ERK were determined by western blot analysis in NSCs after transfection with siR-NC or siR1-Chl1 for 72 h. (B) The expression levels of p-ERK and ERK proteins were measured by western blot analysis in NSCs after treatment with DMSO or Honokiol and cotransfection with LV-miR-10a or LV-NC for 72 h. (C) Representative micrographs of immunofluorescence staining for BrdU (red) with nuclei stained blue with DAPI. Arrows indicate BrdU+ cells. The percentage of BrdU+ cells was significantly greater in the LV-miR-10a+Honokiol group than in the LV-miR-10a+DMSO group. The miR-10a-5p-induced proliferation of NSCs was restored by Honokiol. (D) Representative micrographs of immunofluorescence staining for GFAP (red) with nuclei stained blue with DAPI. The percentage of GFAP+ cells was significantly greater in the LV-miR-10a+Honokiol group than in the LV-miR-10a+DMSO group. The miR-10a-5p-induced differentiation of NSCs was reversed by Honokiol. Scale bar: 50 μm. **P<0.01; ***P<0.001.

Signal transduction induced by Chl1 involves the activation of SRC, PI3 kinase, MEK and ERK, which often positively regulate cell proliferation and cell differentiation [46]. As shown in Figure 6A, siR-Chl1 transfection in NSCs significantly downregulated SRC, PI3K, and ERK1/2 phosphorylation. The ERK agonist Honokiol was used to improve the expression of ERK in cells, and the effect of ERK re-expression on cell function was observed. Compared with those in the LV-NC+DMSO group, the p-ERK44/ERK44 and p-ERK42/ERK42 ratios in the LV-NC+Honokiol group were significantly greater ( *P*=0.0036 and *P*=0.0146, respectively). Compared with those in the LV-miR-10a+DMSO group, the expression levels of p-ERK44/ERK44 and p-ERK42/ERK42 in the LV-miR-10a+Honokiol group were significantly greater ( *P*<0.0001 and *P*=0.0026) ( Figure 6B).

The BrdU assay showed that, compared with that in the LV-NC+DMSO group (66.33%±2.96%), the addition of honokiol after transfection of LV-NC had no significant effect on cell proliferation after ERK overexpression (72.33%±1.45%; *P*=0.1432), and the percentage of BrdU ^+^ cells in the LV-miR-10a+DMSO group was significantly reduced (25.67%±2.33%; *P*=0.0004). However, compared with that in the LV-miR-10a+DMSO group, the percentage of BrdU ^+^ cells in the LV-miR-10a+Honokiol group was significantly greater (55.00%±3.06%; *P*=0.0016), as shown in Figure 6C. These results indicated that the inhibitory effect of miR-10a-5p on cell proliferation could be reversed by the addition of the ERK agonist honokiol, suggesting that ERK is involved in the inhibitory effect of miR-10a-5p on cell proliferation.

The cell immunofluorescence results showed that compared with that in the LV-NC+DMSO group (86.33%±2.96%), the addition of Honokiol posttransfection of LV-NC had a nonsignificant effect on cell differentiation after ERK overexpression (85.67%±2.33%; *P*=0.8683), and the percentage of GFAP ^+^ cells in the LV-miR-10a+DMSO group was significantly decreased (55.67%±2.33%; *P*=0.0012). However, compared with that in the LV-miR-10a+DMSO group, the percentage of GFAP ^+^ cells in the LV-miR-10a+Honokiol group was significantly greater (75.00%±3.06%; *P*=0.0073) ( Figure 6D). These results indicated that the effect of miR-10a-5p on cell differentiation is mediated by regulating the expression of the Chl1 protein and thereby affecting the expression of ERK. Hence, Chl1 seems to inhibit the growth and differentiation of NSCs induced by miR-10a-5p through the ERK1/2 MAPK signaling pathway.

## Discussion

To determine the importance of miRNAs in NTDs, sRNA-seq of RA-induced NTD mouse embryos collected at three different embryonic time points (E8.5, 9.5, and 10.5) was performed, and 41 coexpressed DEmiRNAs were identified. Hierarchical clustering analysis revealed that 41 DEmiRNAs were clustered into five categories. Among them, miR-10a-5p showed the highest expression level and was the most significantly upregulated in NTD embryos compared with that in the normal embryos.

Consistent with the sequencing results, the RT-qPCR results confirmed that the expression levels of miR-10a-5p were significantly increased in RA-induced NTD mouse embryonic NT tissues at E8.5, E9.5 and E10.5, suggesting that miR-10a-5p might be involved in the occurrence of NTDs. Similarly, it has been reported that miR-10a is strongly induced in RA-treated cells. Beveridge *et al*. [48] reported that the expression of miR-10a in SHSY-5Y cells increased after RA treatment. The same results were obtained by H Foley’s study in SK-N-BE and LAN-5 cells [49], which described the upregulation of miR-10a in response to ATRA as a direct consequence of ATRA. It is also hypothesized that ATRA induces a corepressor complex, which binds to the upstream retinoic acid response element of miR-10a and consists of the retinoic acid receptor NCOR2 and histone deacetyltransferase; this complex is replaced by a coactivator complex, leading to chromatin acetylation and concurrent miR-10a transcription. In addition, another study showed that the expression of the hsa-miR-10 family in undifferentiated hESCs was suppressed but was increased significantly in RA-induced hESCs (approximately 95 times) [50]. A previous study identified a mechanism by which retinoid acid receptor-α (RARα) and retinoid X receptor-α (RXRα), as ″directors″ and ″enhancers″, respectively, could enhance RARα binding to the RA-responsive element (RARE) and enhance miR-10a expression [51]. Therefore, the increase in miR-10a-5p expression in RA-induced NTD embryos is not surprising. Nevertheless, further study of the molecular mechanism of miR-10a in the pathogenesis of NTDs is still very important due to the lack of relevant studies.

Furthermore, a functional study showed that miR-10a-5p inhibited cell growth and differentiation. Some previous studies have investigated the role of miR-10a in cell proliferation and differentiation. miR-10a reportedly represses ovarian granulosa cell proliferation by targeting BDNF [52] and promotes microglial cell proliferation by targeting NgR [53]. It also induces neuroblastoma differentiation by targeting NCOR2 [49] and promotes RA-induced differentiation of embryonic stem cells into smooth muscle cells by targeting HDAC4 [54]. These observations indicate that miR-10a plays either a positive or negative regulatory role in the process of cell proliferation and differentiation by targeting different genes and thus controls various cellular pathways in heterogeneous cellular environments, but no studies on this topic in NSCs have been reported. Our study is the first to confirm and report the role of miR-10a-5p in NSCs.

The *Chl1* gene, located at 3p26.3, encodes a cell adhesion molecule of the immunoglobulin superfamily and is a member of the L1 gene family of nerve cell adhesion factors [36]. RT-qPCR and western blot analysis results confirmed that the expression level of Chl1 was significantly decreased in RA-induced NTD mouse embryonic NT tissue. Long *et al*. [47] showed that miR-10a targets *Chl1* and promotes cell growth, migration and invasion in human cervical cancer cells. Our study confirmed that *Chl1* is a target of miR-10a-5p in NSCs, which is consistent with previous results. Functional studies have shown that *Chl1* knockdown inhibits cell growth and differentiation. In addition, the re-expression of *Chl1* without the 3′-UTR reversed the effect of miR-10a-5p on the proliferation and differentiation of NSCs. Huang *et al*. [55] demonstrated that Chl1 is involved in the regulation of proliferation and differentiation of neural progenitor cells (NPCs). Compared with those in the *Chl1* ^+/+^ control group, the proliferation and self-renewal ability of NPCs derived from *Chl1* ^*–*/ *–*^ mice increased, and their differentiation into neurons increased, while the number of GFAP ^+^ astrocytes decreased. The cell proliferation results are contrary to those of this study, which suggests that more research is needed to elucidate the mechanism by which Chl1 affects cell proliferation. Chen *et al*. [56] reported a holoprosencephaly (HPE) foetus with a 7q deletion (including the *SHH* gene) and 3p duplication (including the *Chl1* and *CNTN4* genes) in its chromosome, indicating that these genes may be related to HPE. Our study also showed that miR-10a-5p plays a role in the occurrence of NTDs by inhibiting the expression of the downstream target gene *Chl1*.

Several studies have shown that the function of Chl1 is related to the activation of the SRC-PI3K-MEK-ERK-MAPK signaling pathway [ 46, 57, 58]. The MAPK signaling pathway is an important pathway related to cell proliferation and differentiation and is involved in neural tube development and the occurrence of NTDs. In congenital spina bifida rats, the expression level of MAPK protein was significantly reduced [59]. MAPK is involved in the pathogenesis of anencephaly [60]. Hyperthermia can suppress the phosphorylation of ERK1/2 MAPK, reduce cell proliferation and induce the occurrence of NTDs [ 61, 62]. The expression of SRC/PI3K/ERK in valproic acid-induced NTD mouse embryos was significantly decreased [63]. To investigate whether miR-10a-5p ultimately affects cell proliferation and differentiation by regulating the activity of the MAPK pathway, we performed ERK agonist rescue experiments after LV-miR-10a transfection. Honokiol has neurotropic activity [ 64, 65] and is also an ERK agonist [66]. Our results showed that honokiol supplementation reversed the effects of miR-10a-5p on the proliferation and differentiation of NSCs. These results indicated that miR-10a-5p reduces the expression level of Chl1 and affects cell proliferation and differentiation by inhibiting the MAPK pathway.

In conclusion, this study provided clear evidence of miR-10a-5p upregulation in an RA-induced NTD mouse model, suggesting a possible role for miR-10a-5p in the pathogenesis of RA-induced NTDs. A novel regulatory mechanism of Chl1 in NTDs was also revealed, as it was downregulated by miR-10a-5p through direct targeting of its 3′-UTR. This study also demonstrated that suppression of the ERK/MAPK pathway is accompanied by upregulation of miR-10a-5p expression.

## Supporting information

24078Supplementary_file

## Supplementary Data

Supplementary data is available at *Acta Biochimica et Biophysica Sinica* online.
