# Supplementary material for: Up-regulation of miR-10a-5p expression inhibits the proliferation and differentiation of neural stem cells by targeting Chl1 : Role of miR-10a-5p in NSCs
Source: Acta Biochim Biophys Sin (Shanghai). 2024 Jun 5;56(10):1483–97. doi: 10.3724/abbs.2024078 (PMC11532229; doi:10.3724/abbs.2024078)
Supplement: 24078Supplementary_file [file 24078Supplementary_file.pdf]

Supplementary Table S1. Sequences of primers used for RT-qPCRs

Gene	Accession	Orientation	Primer sequence (5′→3′)
ActB	NM_007393.4	F	GCTCTTTTCCAGCCTTCCTT
		R	AGGTCTTTACGGATGTCAACG
Rgs8	NM_026380.3	F	TGAGGAGTTTGTGGATGTGC
		R	CTGTGGACTTTTCCCTGAGC
Has3	NM_008217.4	F	GTGGGCACCAGTCTGTTTG
		R	CCACTGAACGCGACCTCTG
Elavl3	NM_010487.2	F	GTCAAGAGTCCCCTGTCGC
		R	CTGGGGACAGGTTGTACACG
Lhfpl4	NM_177763.3	F	TCCTTCCTTGCCTTCGTG
		R	AGCCAGACACATCACCTCCT
Rorb	NM_146095.4	F	GACGGCACTGCACAAATTGA
		R	GCAACGGTTCCTGTTGGTTC
Gabrb2	NM_008070.4	F	GTGGTGTGTAGATAGGTGATTTGG
		R	GGGACCGAGATTTCCTCACC
Irs4	NM_010572.2	F	AAATCCAACAATCCGCCTCT
		R	GTATTCTCCCGCTTCCACAA
Chl1	NM_007697.2	F	GTAACCGTCCTTGGTGTTCC
		R	TTGCTGTTGTGGTCATCTCC

Supplementary Table S2. NTDs on E8.5/E9.5/E10.5 after treatment of dams with 28 mg/kg retinoic acid on E7.5

embryonic day	Group	No. of embryos	No. of NTDs	% of NTDs
E8.5	Controls	39	0	0
	RA	35	27	77
E9.5	Controls	37	0	0
	RA	31	25	81
E10.5	Controls	38	0	0
	RA	33	25	76

Supplementary Table S3. miRNAs differentially expressed Bra8.5 vs Brc8.5, Bra9.5 vs Brc9.5 and Bra10.5 vs Brc10.5 NTs

MiRNA[a]	Fold change[b] (log_2Ratio)		
	Bra8.5-vs -Brc8.5	Bra9.5-vs -Brc9.5	Bra10.5-vs -Brc10.5
mmu-miR-10a-5p	1.44	6.07	5.56
mmu-miR-10b-5p	1.54	6.31	6.32
mmu-miR-615-3p	1.89	7.90	4.79
mmu-miR-133a-3p	-2.61	1.60	1.65
mmu-miR-292a-5p	-1.50	-1.20	3.41
mmu-miR-302a-5p	-2.16	-1.42	1.55
mmu-miR-182-5p	-1.26	-1.20	1.65
mmu-miR-295-3p	-1.24	-1.29	1.98
mmu-miR-302b-3p	-1.75	-1.34	1.97
mmu-miR-302d-3p	-1.59	-1.47	1.63
mmu-let-7d-5p	-2.06	1.07	-1.54
mmu-miR-3473d	-2.47	1.90	-2.12
mmu-miR-7663-3p	-5.35	-3.49	-4.97
mmu-miR-344-3p	-3.35	-5.27	-1.82
mmu-miR-9-5p	-1.09	-6.39	-3.23
mmu-miR-128-1-5p	-2.26	-2.64	-2.70
mmu-miR-181a-2-3p	-1.85	-2.82	-2.60
mmu-miR-135a-1-3p	-1.56	-1.83	-2.47
mmu-miR-1933-3p	-1.66	-1.82	-2.22
mmu-miR-410-5p	-1.11	-1.80	-2.58
mmu-miR-6539	-1.22	-2.39	-2.20
mmu-miR-666-3p	-1.23	-2.37	-3.10
mmu-let-7e-5p	-2.55	-1.01	-2.26
mmu-miR-452-5p	-2.77	-1.89	-2.45
mmu-miR-125b-5p	-2.45	-1.40	-1.12
mmu-miR-125a-5p	-1.49	-1.12	-1.19
mmu-miR-181b-5p	-1.30	-1.02	-1.17
mmu-miR-433-5p	-1.73	-1.34	-1.30
mmu-let-7i-5p	-1.60	-1.27	-1.87
mmu-miR-493-5p	-1.23	-1.49	-1.56
mmu-miR-679-5p	-1.31	-1.16	-1.67
mmu-miR-125a-3p	-2.15	-1.70	-1.59
mmu-miR-219a-1-3p	-1.75	-2.31	-1.65
mmu-miR-301b-5p	-1.89	-2.04	-1.98
mmu-miR-135a-5p	-2.20	-2.22	-1.57
mmu-miR-488-5p	-2.22	-2.29	-1.88
mmu-miR-222-3p	-1.42	-2.15	-1.32
mmu-miR-370-5p	-1.41	-2.28	-1.48
mmu-miR-6240	-1.27	-1.96	-1.36
mmu-miR-673-3p	-1.18	-1.77	-1.46
mmu-miR-99b-5p	-1.42	-1.97	-1.17

Abbreviations: NT, neural tube. FDR, false discovery rate. [a]These miRNAs are differentially expressed between RA-treated embryonic NT and normal embryonic NT at E8.5, E9.5 and E10.5 as revealed by miRNA sequencing analysis. [b]Fold changes were calculated based on log_2 of the miRNA expression in Bra/miRNA expression in Brc. MiRNA expression in neural tubes from normal and RA-treated embryos

of E8.5, E9.5 and E10.5 was filtered and the fold change for each miRNA was calculated. Only those miRNAs, which demonstrated a statistically significant(adjusted P<0.05, FDR≤0.001 and the absolute value of $Log_2Ratio \geq 1$) increase or decrease in expression for the Bra8.5-vs-Brc8.5, Bra9.5-vs-Brc9.5 and Bra10.5-vs-Brc10.5 expression comparisons, were included in this table. Note that Bra8.5 vs Brc8.5 means that expression on embryonic day 8.5 of control NT was utilized as the baseline, Bra9.5 vs Brc9.5 means that expression on embryonic day 9.5 of control NT was utilized as the baseline, and Bra10.5 vs Brc10.5 means that expression on embryonic day 10.5 of control NT was utilized as the baseline. Therefore, $\log_2$ (Ratios) below −1.0 indicate a decrease in expression, whereas $\log_2$ (ratios) above 1.0 indicate an increase in expression.

Supplementary Table S4. Differential expression of the predicted target mRNAs of miR-10a-5p in developing NTs

Gene target[a] (symbol)	Fold change[b] (log_2Ratio)			Gene target[a] (symbol)	Fold change[b] (log_2Ratio)		
	Bra8.5-vs-Brc8.5	Bra9.5-vs-Brc9.5	Bra10.5-vs-Brc10.5		Bra8.5-vs-Brc8.5	Bra9.5-vs-Brc9.5	Bra10.5-vs-Brc10.5
Hoxa1	0.98	5.22	4.26	Cyth1	-0.14	0.01	-0.46
Hoxd10	1.55	10.13	4.25	**Has3**	**-0.57**	**-0.41**	**-1.05**
Ltbp1	0.30	0.56	0.46	Celf2	-0.20	0.03	-0.60
Sh3d19	0.22	0.52	0.25	Klhl29	-0.22	-0.14	0.48
Gata6	0.41	3.36	2.98	**Elavl3**	**-0.36**	**-0.57**	**-1.10**
Hoxa3	1.64	3.87	6.47	Mtf1	-0.21	-0.36	-0.65
Igsf1	0.42	0.31	1.09	Inhbb	0.03	-0.03	-0.88
Pea15a	-0.31	-0.57	-0.44	Tmem183a	-0.25	-0.61	-0.61
Elavl2	-0.10	-0.82	-0.74	**Lhfpl4**	**-0.07**	**-1.08**	**-2.00**
Hoxb3	1.50	3.12	4.62	Sobp	-0.66	0.41	0.21
Slc25a1	0.19	0.59	0.38	Ankfy1	-0.01	-0.24	-0.50
Ppara	-0.19	-0.70	-0.03	Fbf1	-0.10	0.11	-0.11
Trim39	-0.22	-0.48	-0.49	Zfp827	0.16	0.25	-0.36
E2f7	-0.04	-0.57	-0.47	Mapre1	-0.10	-0.58	-0.60
Nr5a2	0.46	2.64	1.38	**Rorb**	**0.95**	**-0.90**	**-1.74**
Rhpn2	0.51	0.74	1.39	**Gabrb2**	**0.92**	**-0.97**	**-2.18**
Zfp367	-0.21	-0.07	-0.04	Hspa12a	-0.04	0.49	-0.13
Cecr6	-0.24	-0.39	-0.66	**Irs4**	**-1.50**	**-3.31**	**-2.28**
Tbx5	-0.87	2.30	-0.03	Shisa7	-0.13	0.19	-0.56
Baz1b	-0.01	-0.52	-0.61	Spag9	0.02	0.13	-0.38
Palm2	-1.12	-0.01	0.50	Nav1	-0.11	-0.86	-0.51
Camk2b	-0.16	1.82	1.39	Pten	-0.15	-0.55	-0.37
Nfasc	-0.65	-0.43	-0.25	E130309D1	0.27	-0.67	-0.15
Pcdh10	-0.65	-0.48	-0.25	Baz2b	0.10	0.07	-0.37
**Rgs8**	**-1.52**	**-1.44**	**-1.82**	Lix1l	0.05	0.26	-0.07
Purg	0.06	-0.18	-0.20	Tnrc6b	0.10	0.10	-0.49
Nr2c2	-0.01	-0.44	-0.67	E2f3	-0.14	-0.67	-0.70
Bbx	0.53	0.62	0.84	**Chl1**	**-0.46**	**0.53**	**-1.41**
Mapkbp1	0.27	-0.39	-0.34	Npas3	0.30	0.73	-0.65
Prtg	0.03	0.22	1.79	Actg1	-0.14	0.58	0.42
Rc3h2	-0.08	-0.40	-0.83	Srsf1	-0.15	-0.64	-0.64
Sertad4	0.16	-0.36	-0.68	Tmem170b	0.35	0.16	0.11
Myt1l	-0.43	-0.12	-0.86	Scn3a	-0.79	2.28	-0.70
Epha8	0.24	-0.33	-0.46				

Abbreviations: NT, neural tube. FDR, false discovery rate. [a]These 67 predicted target genes of miR-10a-5p were obtained by a combined analysis of sRNA-seq and mRNA-seq data by MAGIA[2], which was used to analyze the target predictions, miRNA and gene expression data. [b]Fold changes were calculated based on log_2 of the mRNA expression in Bra/mRNA expression in Brc. Gene expression in

neural tubes from normal and RA-treated embryos of E8.5, E9.5 and E10.5 was filtered and the fold change for each gene was calculated. Note that Bra8.5 vs Brc8.5 means that expression on embryonic day 8.5 of control NT was utilized as the baseline, Bra9.5 vs Brc9.5 means that expression on embryonic day 9.5 of control NT was utilized as the baseline, and Bra10.5 vs Brc10.5 means that expression on embryonic day 10.5 of control NT was utilized as the baseline. A combination of adjusted $P < 0.05$, FDR $\leqslant 0.001$ and the absolute value of $Log_2 Ratio \geq 1$ was used as the threshold to determine the significance of gene expression difference. Therefore, log_2 (Ratios) below -1.0 indicate a decrease in expression (red font), whereas log_2 (Ratios) above 1.0 indicate an increase in expression (green font). Gene (mRNA) targets whose expressions were decreased on at least one time point on E9.5 or E10.5 are presented in bold letters with a gray background.

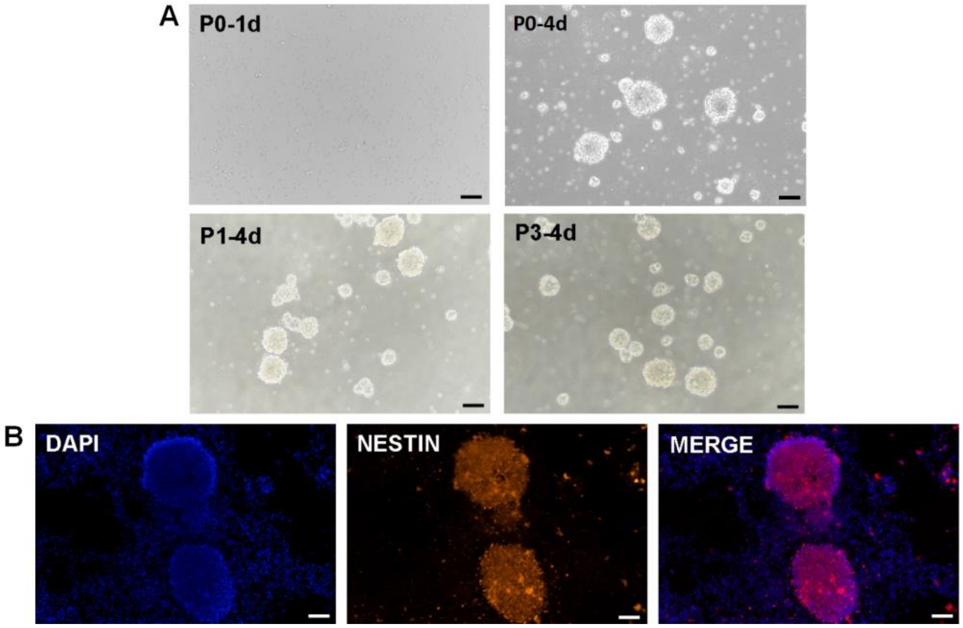

Supplementary Figure S1. Culture and nestin identification of NSCs *in vitro* (A) Cell morphology of NSCs on day 1 (P0-1d) and day 4 (P0-4d) of the primary generation, day 4 (P1-4d) of the first generation, day 4 (P3-4d) of the third generation. (B) After the third generation of NSCs culture, the nerve spheres were identified by immunofluorescence staining. Scale bar: 100 μm

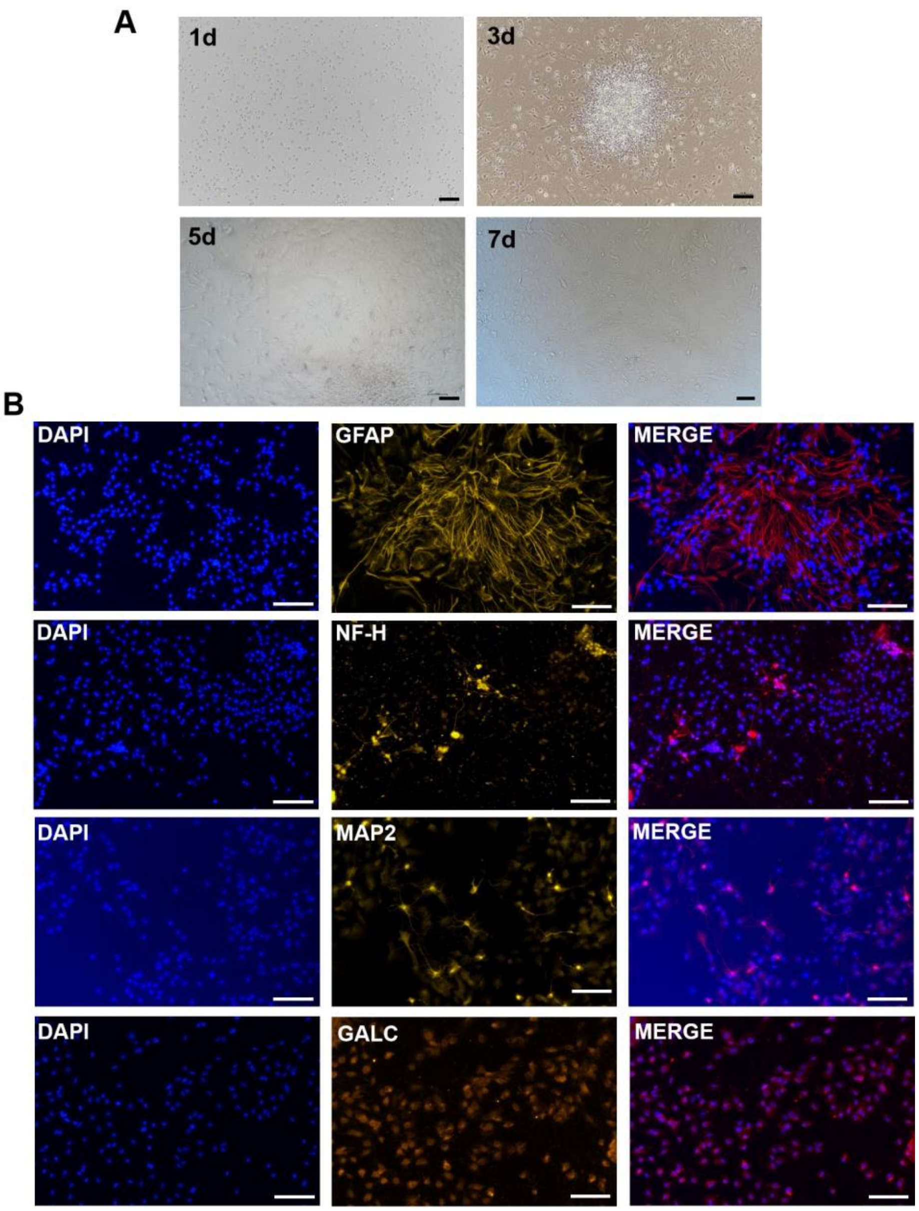

Supplementary Figure S2. Serum-induced culture and differentiation identification of NSCs *in vitro*
(A) Cell morphology of NSCs after 1, 3, 5 and 7 days of induced differentiation culture. (B) After 7 days of NSCs differentiation, immunocytochemical staining was performed with GFAP, NF-H, MAP2 and GALC. GFAP is the expression marker of astrocytes, NF-H and MAP2 are the expression markers of neurons, and GALC is the expression marker of oligodendrocytes, respectively. Scale bar: 100 μm

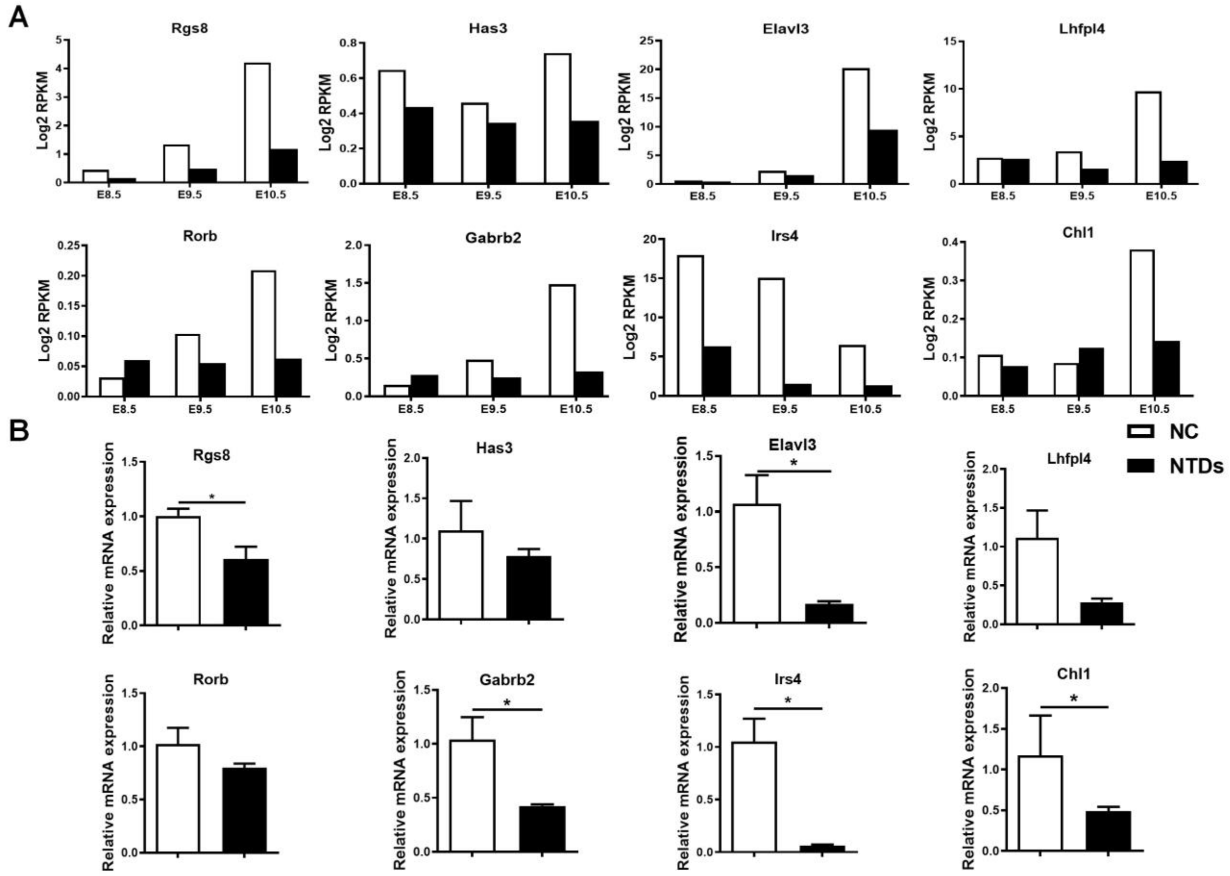

Supplementary Figure S3. Expressions of 8 genes in mice treated with RA (A) Analysis of mRNA-seq results of eight genes at E8.5, E9.5 and E10.5. (B) Relative expression of 8 genes were measured by RT-qPCR at E8.5, E9.5 and E10.5 (*P<0.01).
